# ^1^H NMR-based metabolomics of paired tissue, serum and urine samples reveals an optimized panel of biofluids metabolic biomarkers for esophageal cancer

**DOI:** 10.3389/fonc.2023.1082841

**Published:** 2023-01-23

**Authors:** Ting Ouyang, Changchun Ma, Yan Zhao, Wei Ye, Jiayun Zhao, Rongzhi Cai, Huanian Zhang, Peie Zheng, Yan Lin

**Affiliations:** ^1^ Radiology Department, Second Affiliated Hospital, Shantou University Medical College, Shantou, Guangdong, China; ^2^ Radiology Department, People’s Hospital of Leshan, Leshan, Sichuan, China; ^3^ Radiation Oncology, Affiliated Tumor Hospital, Shantou University Medical College, Shantou, Guangdong, China

**Keywords:** biomarker, biofluids, esophageal squamous cell carcinoma, 1H NMR-based metabolomics, predictive nomogram

## Abstract

**Introduction:**

The goal of this study was to establish an optimized metabolic panel by combining serum and urine biomarkers that could reflect the malignancy of cancer tissues to improve the non-invasive diagnosis of esophageal squamous cell cancer (ESCC).

**Methods:**

Urine and serum specimens representing the healthy and ESCC individuals, together with the paralleled ESCC cancer tissues and corresponding distant non-cancerous tissues were investigated in this study using the high-resolution 600 MHz 1H-NMR technique.

**Results:**

We identified distinct 1H NMR-based serum and urine metabolic signatures respectively, which were linked to the metabolic profiles of esophageal-cancerous tissues. Creatine and glycine in both serum and urine were selected as the optimal biofluids biomarker panel for ESCC detection, as they were the overlapping discriminative metabolites across serum, urine and cancer tissues in ESCC patients. Also, the were the major metabolites involved in the perturbation of “glycine, serine, and threonine metabolism”, the significant pathway alteration associated with ESCC progression. Then a visual predictive nomogram was constructed by combining creatine and glycine in both serum and urine, which exhibited superior diagnostic efficiency (with an AUC of 0.930) than any diagnostic model constructed by a single urine or serum metabolic biomarkers.

**Discussion:**

Overall, this study highlighted that NMR-based biofluids metabolomics fingerprinting, as a non-invasive predictor, has the potential utility for ESCC detection. Further studies based on a lager number size and in combination with other omics or molecular biological approaches are needed to validate the metabolic pathway disturbances in ESCC patients.

## Introduction

Esophageal cancer (EC) is the sixth leading cause of cancer death and the eighth most common cancer worldwide, with a 5-year survival rate of only 15%-25% (1). In China, the EC incidence currently ranks first in the world, among which more than 90% are esophageal squamous cell carcinoma (ESCC) (2), and Chaoshan region of Guangdong Province is the only coastal area with a high incidence of ESCC in China (3). ESCC often presents with progressive dysphagia, most of which have advanced to a later stage with significantly lower 5-year survival rates (4). Hence, early diagnosis of ESCC is of great significance for optimized disease management (5). Endoscopic esophageal biopsy carries the risks of bleeding, tearing and perforation, which compromises its wider applicability for ESCC early diagnosis (6, 7). The sensitivity and specificity of serum tumor markers such as CA199 and CA125 for ESCC diagnosis are not high enough (<50%) (8). Esophageal barium meal and CT imaging involve in radiation hazards and are prone to miss small EC lesions (6, 7). Recently, liquid biopsy assays based on genetic alterations, such as serum DNA, microRNA and others, have been actively investigated. However, they are too expensive and have low diagnostic sensitivity, such reducing the ESCC screening reliability (9–11). Due to the lack of non-invasive, simple, and accurate screening biomarkers, the early diagnosis rate of ESCC in China is extremely low.

Metabolism reprogramming is considered a hallmark of cancer. Each cancer has its unique metabolic characteristics, which are closely related to the occurrence and progression of the tumor, thus providing a biochemical basis to define disease biomarkers for early cancer diagnosis. Nuclear magnetic resonance (^1^H NMR)-based metabolomics provides simple, efficient and inexpensive technical support to discover potential cancer metabolic biomarkers for ESCC detection, mainly focusing on the analysis of urine, serum and tissue samples (12–15), respectively. Tissue is the lesion site of cancer, which contains global biological metabolic information at the levels of metabolic enzymes and metabolite levels. Therefore, *in situ* targeted detection of cancer tissue is the direct method to identify tumor-specific metabolic biomarkers. However, the tissue sampling process is invasive and prone to missing small lesions or sampling errors. Recently, we identified distinct NMR-based serum and urine metabolic signatures, respectively, which were linked to the metabolic profiles of esophageal-cancerous tissues (16, 17). However, single serum or urine metabolism can only partially characterize the metabolic characteristics of the body. Urinary metabolism is mainly derived from the metabolic state of the urinary system, while blood metabolism is the phenotype of the interaction between gut microbes and the host, including the circulatory system, endocrine system and immune system, etc. If an optimal metabolic panel through combining the biomarkers in serum and urine can be constructed, it is possible to achieve more comprehensive metabolic information of ESCC, so as to improve the efficiency of ESCC non-invasive screening.

The aim of this study was to establish an optimized metabolic profile to improve the non-invasive diagnosis of ESCC by combining serum and urine biomarkers that could reflect the malignancy of tumor tissues. Urine and serum specimens representing the healthy controls and ESCC individuals were examined using the high-resolution 600 MHz ^1^H NMR technique. Meanwhile, the paralleled patient-matched metabolites of ESCC cancer tissue together with their non-cancerous mucosa in the distant areas were investigated, which were used as references to determine biofluids metabolic biomarkers. Pattern recognition was applied on NMR processed data to acquire detailed metabolic information. Finally, an optimized panel of differential metabolites based on serum and urine metabolic biomarkers were selected and then constructed as a predictive nomogram to predict the risk of ESCC occurrence using multiple regression analysis.

## Materials and methods

### Clinical samples

The Ethical Review Board of Shantou University Medical College approved the metabolic analysis of the samples in this study. This study involved 70 ESCC individuals and 70 healthy controls (HCs) with signed informed consent. ESCC patients were matched with HCs according to age and gender. The experimental group included 50 ESCCs patients who underwent esophagectomy and provided their esophageal cancer tissues (ECT), distant non-cancerous tissues (DNT; about 5 cm away from the cancer tissue and hematoxylin and eosin staining proved no malignancy), along with preoperative serum and urine samples. Control serum and urine samples were obtained from 50 HCs with no history of associated gastrointestinal problems. As for the validation group, serum and urine obtained from 20 ESCCs and 20 HCs were collected for verifying the predictive ability of the metabolic model in the experimental group. All samples were stored in a low-temperature refrigerator (−80°C) until further metabolic extraction. ESCC patients who received radiotherapy or preoperative chemotherapy were excluded. **Table 1** summarizes the clinical characteristics of the subjects included in this study.

**Table 1:** Summary of the clinical and demographic characteristics of the study subjects.

	test set		validation set	
characteristics	ESCC	health	ESCC	health
Number	50	50	20	20
Gender (male/female)	29/21	25/25	12/8	10/10
Age (years) medium	57	55	57	50
<40	9	6	3	4
40-49	11	11	5	6
50-59	18	19	5	5
>60	12	14	7	5
BMI (kg/m2)	27.3 ± 4.7	29.3 ± 5.3	27.1 ± 4.2	28.8 ± 4
Cancer stage				
Stage I/II	32		13	
Stage III/IV	18		7	
CEA (ng/mL)				
Positive	29	N/A	12	N/A
Negative	21	N/A	8	N/A
CA199 (U/mL)				
Positive	24	N/A	12	N/A
Negative	26	N/A	8	N/A
Tumor location				
cervical	1		1	
upper thoracic	8		3	
middle thoracic	27		9	
lower thoracic	14		7	

N/A, normal/absence.

### Sample preparation

Esophageal tissue, serum and urine were pre-processed according to our previous publications (16, 17). Basically, frozen tissue samples (about 300 mg) were thawed at 25°C, then sliced and homogenized at 16,000 rpm for 80 seconds in a solution consisting of 0.6 mL distilled water and 1.2 mL methanol. Subsequently, chloroform and distilled water were further added with 1.2mL each and the samples were vortexed for 60 seconds. Next, samples were incubated for 15 mins on ice and then centrifugated for 5 mins at 2000 rpm. The resulting supernatant was washed with nitrogen and evaporated, followed by incubation under vacuum for at least 18 h. Finally, the lyophilized powder was dissolved further in PBS/D2O buffer solution (550 μL, 0.1 M, pH 7.4) containing TSP/D_2_O stock solution (50 μL). After centrifugation of the mixture for 5 mins at 10,000 rpm, 500 μL of the supernatant was transferred to an NMR tube (5 mm in size) for ^1^H NMR spectroscopy. As for frozen serum and urine samples preparation, they were thawed at 25°C, respectively. After adding 200 μL PBS/D_2_O buffer to 400 μL of each urine and serum sample, respectively, the mixtures were then swirled for 60 seconds and centrifuged at 10,000 rpm for 5 minutes. Finally, ^1^H NMR analysis was carried out by using 500 μL of the serum supernatant. While for urine, 50 μL of TSP/D_2_O stock solution was added to the supernatant before ^1^H NMR spectral acquisition.

### ^1^H NMR spectral analysis

^1^H NMR spectra of all samples were acquired by using a Bruker Avance NMR spectrometer (Bruker Corporation, Germany) operating at 600MHz and 298K. NOESYPR1D pulse sequence was used to detect the ^1^H-NMR spectrum of esophageal tissues and urine samples, and the acquisition parameters were as follows: 90° Pulse Width, 14.1 μs; Relaxation Delay (RD), 4.0 s; Echo Time (TE), 70 ms; Spectral Width (SW), 12335 Hz; number of points (TD), 32,768; number of scans (NS), 64; acquisition time (AQ), 2.66 s. By using the 1D CPMG pulse sequence, serum ^1^H NMR spectra were recorded, and the acquisition parameters were as follows: 90° pulse width, 12.2μs; RD, 4.0 s; TE, 70 ms; SW, 12019 Hz; TD, 32,768; NS, 64 and AQ, 2.73 s. Irradiating a peak of water between RD and t*_m_* to achieve water suppression.

### ^1^H NMR spectral processing

Before Fourier Transformation, all FIDs were multiplied by a 1 Hz line-broadening to enhance the SNR. The spectrum was then manually phase adjusted, followed by baseline correction and frequency alignment with reference to the 0.0 ppm TSP signal. In order to minimize the complexity of the spectrum data, the spectral regions ofδ9.0-0.5 were divided into buckets with 0.004 ppm width, along with the removal of the residual water (4.5 to 5.5 ppm). Each bucket was normalized to the total integral of the spectrum to reduce concentration differences between samples while improving sample comparability.

### Pattern recognition

The normalized integral data were uploaded into SIMCA-P14.1 software (Umetrics AB, Umeå, Sweden) to perform pattern recognition based on previous publications (16, 17), to separate ESCC patients’ urine and serum samples from those of HCs, as well as ESCC cancer tissues from their DNT samples. All NMR spectral datasets were first mean-centered, and then performed principal component analysis (PCA) to visualize samples` outliers or clusters. Following PCA, the spectral data were treated with orthogonal partial least squares-discriminant analysis (OPLS-DA) so that the experimental groups could be better distinguished. OPLS-DA was first established by Kirwan and Johansson based on PLS algorithm in 2002 (18). R^2^Y values (ranging from 0 to 1) and Q^2^ values (ranging from negative to 1) were used to assess the model quality. A value of R^2^Y equal to 1 indicates that the model explains 100% of the variance, and a value of Q^2^ close to 1 means a reliable prediction in cross-validation. By using a permutation test of 400-times, the quality of OPLS-DA model was validated, where R^2^Y represents the degree of fit between the model and the data, and Q^2^ indicates how strong the predictive power of the model is, with a Q^2^ greater than 0.5 considered “good” and a Q^2^ greater than 0.9 deemed “excellent”. The peak selection coefficients were the VIP (variable importance of projection) from the OPLS-DA model. By integrating the area under the peak, the relative levels of the chosen peaks with VIP greater than 1 were quantified. The major metabolites in the profile were identified according to the available literature (19–21) and data from the Human Metabolome Database. Metabolites with VIP ≥ 1 and significant differences in abundance among study groups were classified as candidate biomarkers, and differences were expressed as mean fold difference (FD), which was obtained by dividing the relative abundance of metabolites in ESCC and by that in their corresponding controls.

### Analysis of TCGA database

The RNAseq data in TCGA and GTEx TPM format were processed uniformly by Toil process from UCSC XENA (https://xenabrowser.net/datapages/). The expression of key genes related to the important metabolic pathway in ESCC cancer tissues were extracted from TCGA-ESCA (esophageal cancer) and GTEx databases. The expression of RNAseq data in TPM (transcripts per million reads) format and log_2_ transformation was compared between samples.

### Statistical analysis

The normality test was conducted for all data and the corrected *p* value was obtained by using the Benjamini-Hochberg method, that is, the False discovery rate (FDR) corrections. False discovery rate (FDR) corrections were calculated to account for the multiple comparisons that often occur in metabolomics studies. In order to evaluate the diagnostic performance of metabolic biomarkers, ROC analysis was conducted and the area under the curve (AUC) was calculated using SPSS 16.0, with AUC≥0.8 indicating good diagnostic ability. Spearman correlation analysis was used to assess the association of potential biomarkers in the urine, serum and cancer tissues of ESCC patients using correlation coefficient (r). The cutoff value of the correlation coefficient r was determined according to the sample size. Correspondingly, |r|≥0.28 indicates a significant correlation between two kinds of metabolites, based on the sample size of 50 in the experimental group.

## Results

### Metabolic profiles and pattern recognition of esophageal cancer tissue, serum and urine samples

**Figure 1:**
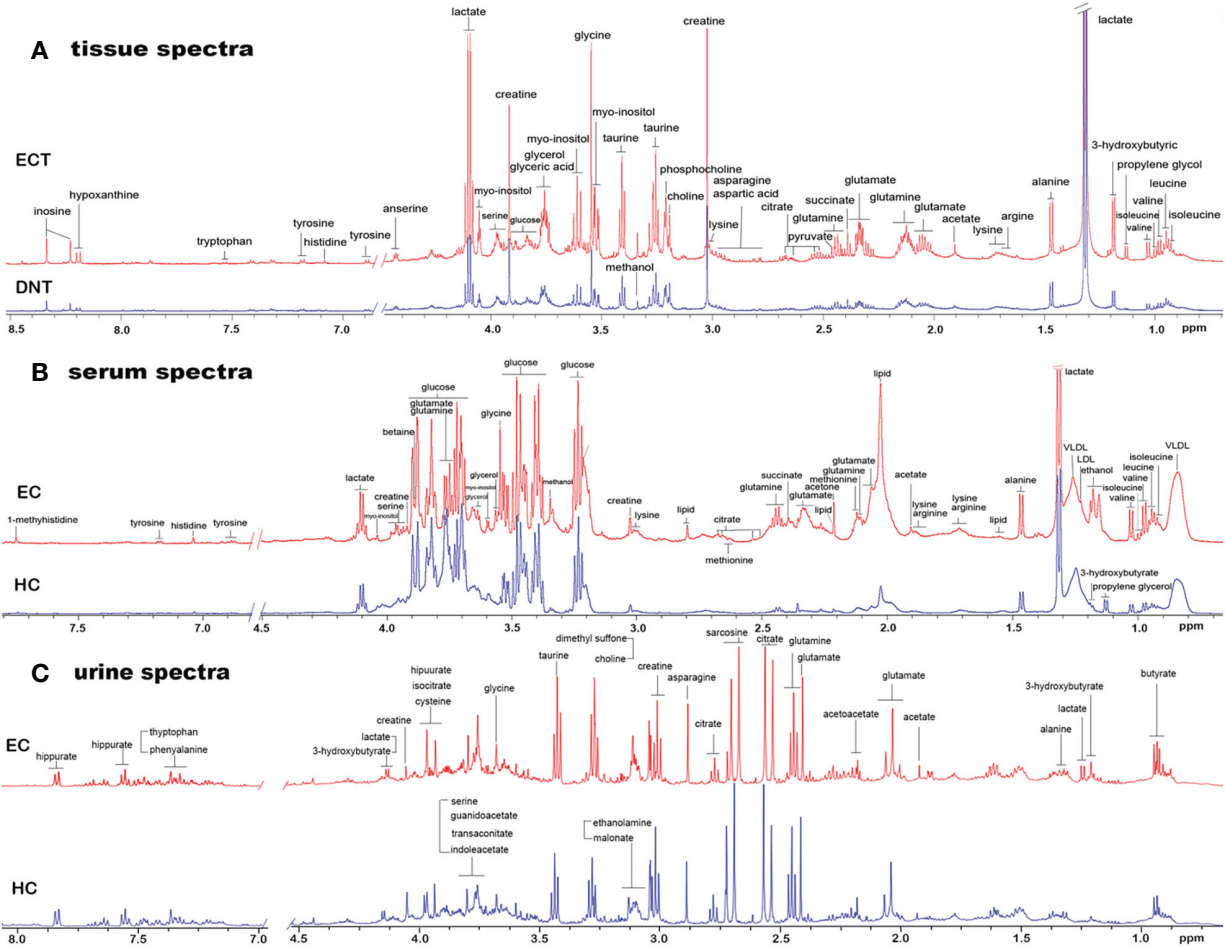
Representative 1D 1H NMR spectra of tissue, serum and urine from EC patients and controls. **(A)** Representative 600MHz NOESYPR1D 1H NMR spectra of esophageal tissue extracts from ECT and DNT. **(B)** Representative 600MHz CMPG 1H NMR spectra of serum from EC and HC. **(C)** Representative 600MHz NOESYPR1D 1H NMR spectra of urine EC and HC. ECT, esophageal cancer tissue; DNT, distal noncancerous tissue; EC, esophgeal cancer; HC, healthy control.

**Figure 2:**
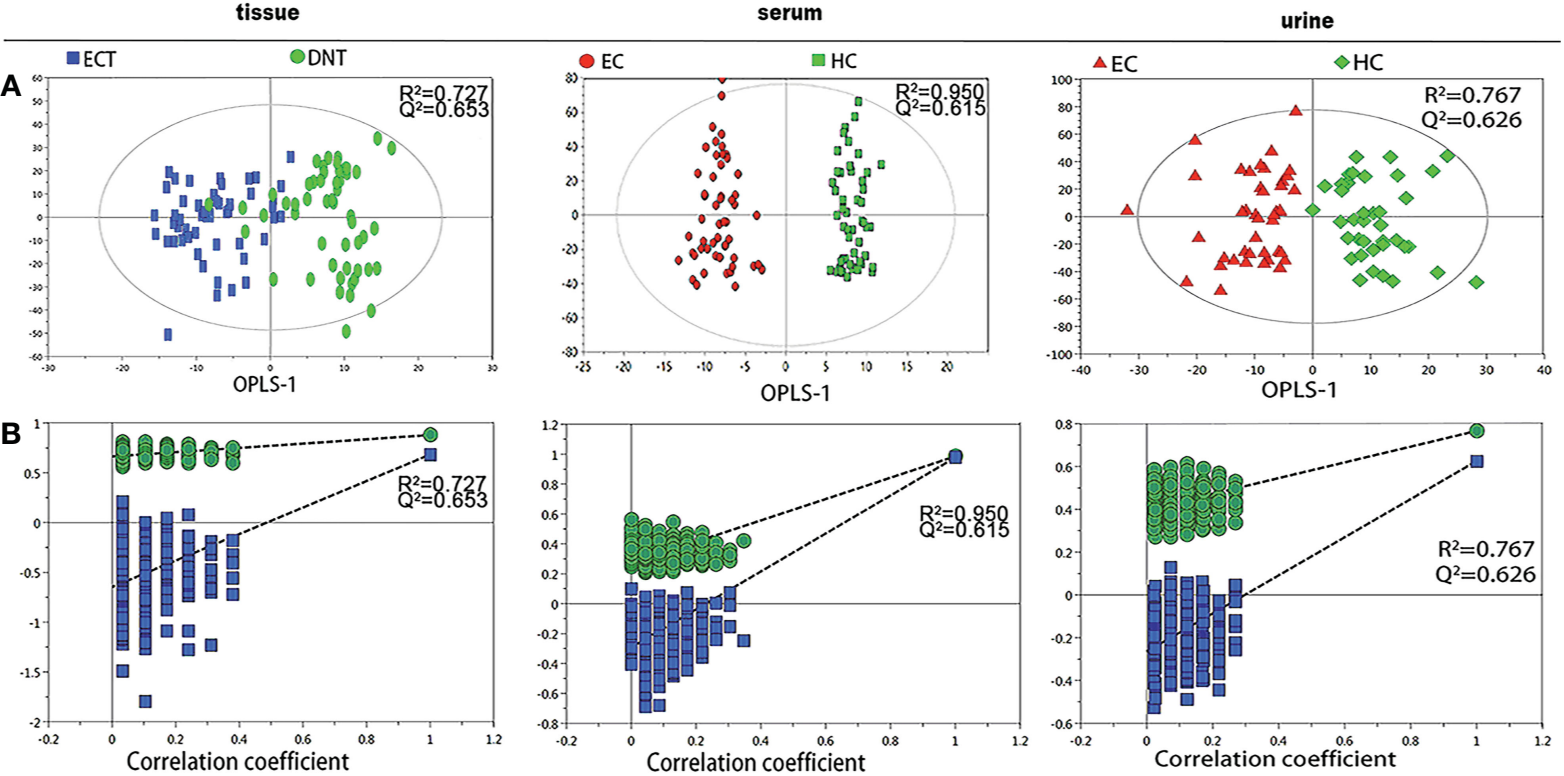
Pattern recognition analysis of ^1^H-NMR tissue, serum and urine spectra between ESCC and their respective controls. **(A)** OPLS-DA scores plot depicting the difference between experimental groups; **(B)** Statistical validation of the corresponding OPLS-DA model by permutation analysis (400 times).

**Figure 1** shows representative ^1^H-NMR spectra of ECT and DNT, as well as those of serum and urine samples from ESCC patients and HCs. In the pattern recognition of the processed ^1^H NMR spectra from tissue extracts, serum and urine in ESCC patients and those from their corresponding controls, the score plots revealed a differential clustering alongside the first principal component direction (**Figure 2A**). The OPLS-DA model was verified by 400 times permutation analysis (**Figure 2B**): DNT vs ECT: R^2^Y= 0.727, Q^2 =^ 0.653; normal vs ESCC serum: R^2^Y= 0.950, Q^2 =^ 0.615; normal vs ESCC urine: R^2^Y= 0.767, Q^2 =^ 0.626, indicating there was no overfitting in OPLS-DA model.

Under such criteria (VIP > 1, fold change ≥1.1 or ≤0.9, *p*<0.05), a number of potential metabolite biomarkers in the tissue, serum and urine in ESCC patients were observed, as shown in **Table 2**. ESCC tumor tissues exhibited higher levels of leucine, isoleucine, arginine, valine, lysine, glutamate, glutamine, asparagine, choline, phosphocholine and glycine, together with lower levels of lactate, alanine, pyruvate, citrate, creatine, taurine, myo-inositol and glucose, as compared to their corresponding DNT. Twelve serum metabolites were observed to have altered notably in ESCC patients in comparison with healthy controls, including elevated levels of glutamate, glutamine, glycerol, myo-inositol and glucose, and reduced levels of leucine, isoleucine, lactate, lipid, lysine, creatine and glycine. Compared with healthy controls, urine samples in ESCC showed increased amounts of acetoacetate, indoleacetic acid, cis-aconitate, and reduced levels of alanine, creatine, ethanolamine, glycine, glucose, cysteine and hippuric acid. A few overlapping characteristic metabolites across serum, urine and tissue samples in ESCC patients were observed from our paralleled studies, including glycine and creatine.

**Table 2:** Potential biomarkers for discriminating samples from ESCC patients and HCs.

Metabolites	Chemical shift(ppm)	Fold difference		AUC (95%CL)	Related metabolic pathways
		EC/HCs	P value		
Isoleucine	0.93-0.95	1.14↑	0.015	0.686 (0.548-0.824)	Amino acid metabolism
	1.01-1.03	1.25↑	<0.001	0.826 (0.719-0.933)
Leucine	0.95-0.98	1.27↑	<0.001	0.841 (0.736-0.945)	Amino acid metabolism
Valine	0.99-1.00	1.19↑	0.001	0.765 (0.643-0.886)	Amino acid metabolism
	1.03-1.06	1.22↑	<0.001	0.775 (0.656-0.894)
Arginine	1.65-1.78	1.14↑	0.001	0.719 (0.586-0.829)	Amino acid metabolism
Lysine	3.01-3.03	1.06↑	<0.001	0.749 (0.618-0.854)	Amino acid metabolism
Glutamate	2.02-2.10	1.24↑	<0.001	0.847 (0.728-0.928)	Glutaminolysis, gluconeogenesis
	2.31-2.40	1.31↑	<0.001	0.895 (0.787-0.960)
Asparagine	2.79-2.99	1.15↑	<0.001	0.735 (0.603-0.842)	Amino acid metabolism
Choline	3.20-3.21	1.41↑	<0.001	0.795 (0.669-0.890)	Choline metabolism
Glycine	3.56-3.57	1.35↑	<0.001	0.860 (0.743-0.937)	Amino acid metabolism
Glutamine	2.10-2.19	1.09↓	0.003	0.710 (0.576-0.822)	Glutaminolysis, gluconeogenesis
	4.09-4.14	0.95↓	0.038	0.653 (0.516-0.773)
Alanine	1.47-1.50	0.83↓	<0.001	0.738 (0.606-0.845)	Amino acid metabolism
Pyruvate	2.47-2.48	0.91↓	0.039	0.652 (0.515-0.772)	TCA cycle activity
Citrate	2.65-2.66	0.91↓	0.006	0.692 (0.557-0.807)	TCA cycle activity
Creatine	3.93-3.94	0.90↓	0.005	0.704 (0.570-0.817)	Energy metabolism
Taurine	3.41-3.44	0.91↓	0.041	0.652 (0.515-0.772)	Amino acid metabolism
Myo-inositol	3.61-3.65	0.81↓	<0.001	0.768 (0.639-0.869)	Amino acid metabolism
Glucose	3.80-3.91	0.86↓	0.011	0.681 (0.546-0.798)	Energy metabolism
Glutamate*	2.29-2.36	2.44↑	0.004	0.672 (0.548-0.796)	Glutaminolysis, gluconeogenesis
Glycerol*	3.56-3.64	1.21↑	<0.001	0.788 (0.682-0.872)	Fatty acid metabolism
Myo-inositol*	3.59-3.61	1.66↑	<0.001	0.798 (0.917-0.998)	Fatty acid metabolism
Glucose*	3.22-3.91	1.44↑	<0.001	0.978 (0.917-0.998)	Energy metabolism
Glutamine*	3.76-3.78	1.96↓	<0.001	0.955 (0.890-0.987)	Glutaminolysis, gluconeogenesis
	2.41-2.46	1.52↓	<0.001	0.905 (0.825-0.956)	
Lipid*	1.53-1.60	0.56↓	<0.001	0.922 (0.848-0.968)	Fatty acid metabolism
	2.72-2.82	0.76↓	<0.001	0.864 (0.777-0.926)	
Lysine*	2.99-3.02	0.61↓	<0.001	0.953 (0.881-0.988)	Amino acid metabolism
Creatine*	3.02-3.04	0.79↓	<0.001	0.820 (0.727-0.893)	Energy metabolism
	3.91-3.92	0.62↓	<0.001	0.956 (0.891-0.988)	
Glycine*	3.54-3.55	0.83↓	<0.001	0.780 (0.673-0.865)	Amino acid metabolism
Acetoacetate#	2.26-2.30	1.44↑	<0.001	0.721 (0.609-0.815)	Fatty acid metabolism, TCA cycle
Cis-aconitate#	3.40-3.48	1.30↑	<0.001	0.714 (0.602-0.810)	TCA cycle, glyoxylate metabolism
Indoleacetate#	3.70-3.73	1.25↑	0.002	0.684 (0.571-0.784)	Amino acid metabolism
Alanine#	1.47-1.51	0.74↓	<0.001	0.744 (0.635-0.835)	Amino acid metabolism
Creatine#	3.05-3.09	0.74↓	<0.001	0.707 (0.596-0.802)	Energy metabolism
	4.06-4.13	0.73↓	<0.001	0.713 (0.602-0.807)	
Ethanolamine#	3.11-3.19	0.94↓	<0.001	0.734 (0.624-0.827)	Fatty acid metabolism
Glycine#	3.57-3.59	0.87↓	0.010	0.659 (0.544-0.761)	Amino acid metabolism
Glucose#	3.75-3.85	0.90↓	0.015	0.651 (0.536-0.754)	Energy metabolism
Cysteine#	3.95-3.99	0.78↓	<0.001	0.808 (0.704-0.887)	Amino acid metabolism
Hippurate#	3.94-4.03	0.83↓	<0.001	0.790 (0.686-0.873)	Gut microflora metabolism
	7.52-7.67	0.40↓	<0.001	0.709 (0.598-0.804)	
	7.82-7.86	0.44↓	<0.001	0.295 (0.594-0.800)	

Metabolites without label noted tissue biomarkers; metabolites labeled with “*” and “#” noted serum and urine biomarkers, respectively. The up/down arrow indicates that the relative content of differential metabolites in tissue, serum or urine of esophageal cancer is higher/lower than that of the control group.

### Correlation analysis across serum, urine and tissue metabolites in ESCC patients

**Figure 3:**
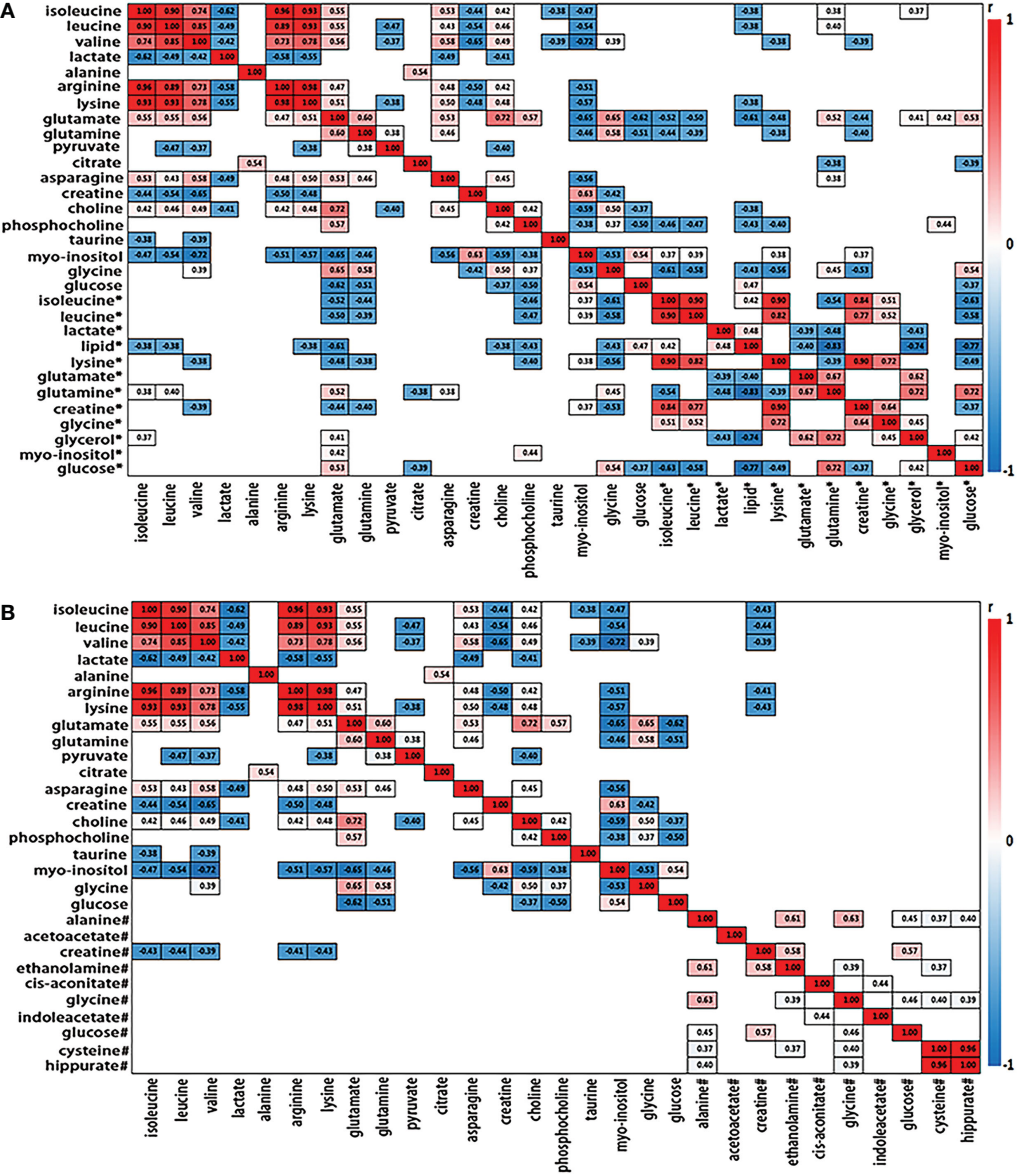
Heat map color-coded based on the strength of spearman correlation coefficients of metabolites identified as important in tumor vs serum discrimination **(A)** and urine discrimination **(B)** in test group. The cutoff values of |r| > 0.28 and *p*< 0.05 have been used (n=50). The metabolites used are given in **Table 1**. Red boxes indicated positive associations and blue boxes indicated negative associations. Metabolites without label noted tissue biomarkers; metabolites labeled with “*” and “#” noted serum and urine biomarkers, respectively.

We further analyzed the metabolic profiling association between serum and tissue biomarkers, as well as between urine and tissues biomarkers, being plotted as correlation heat maps (**Figures 3** **A, B**), which showed that changes of serum isoleucine, leucine, lysine, glutamine, creatine, glycerol, myo-inositol and glucose in ESCC patients were closely related to the changes of most metabolites in ESCC cancer tissues (|r| >0.3, p<0.05). Alterations of urine creatine in ESCC patients were negatively associated with the changes in isoleucine, leucine, valine, arginine and lysine in ESCC cancer tissues (|r| >0.3, p<0.05).

### Selection of an optimal biomarker panel based on serum and urine metabolic characteristics

**Figure 4:**
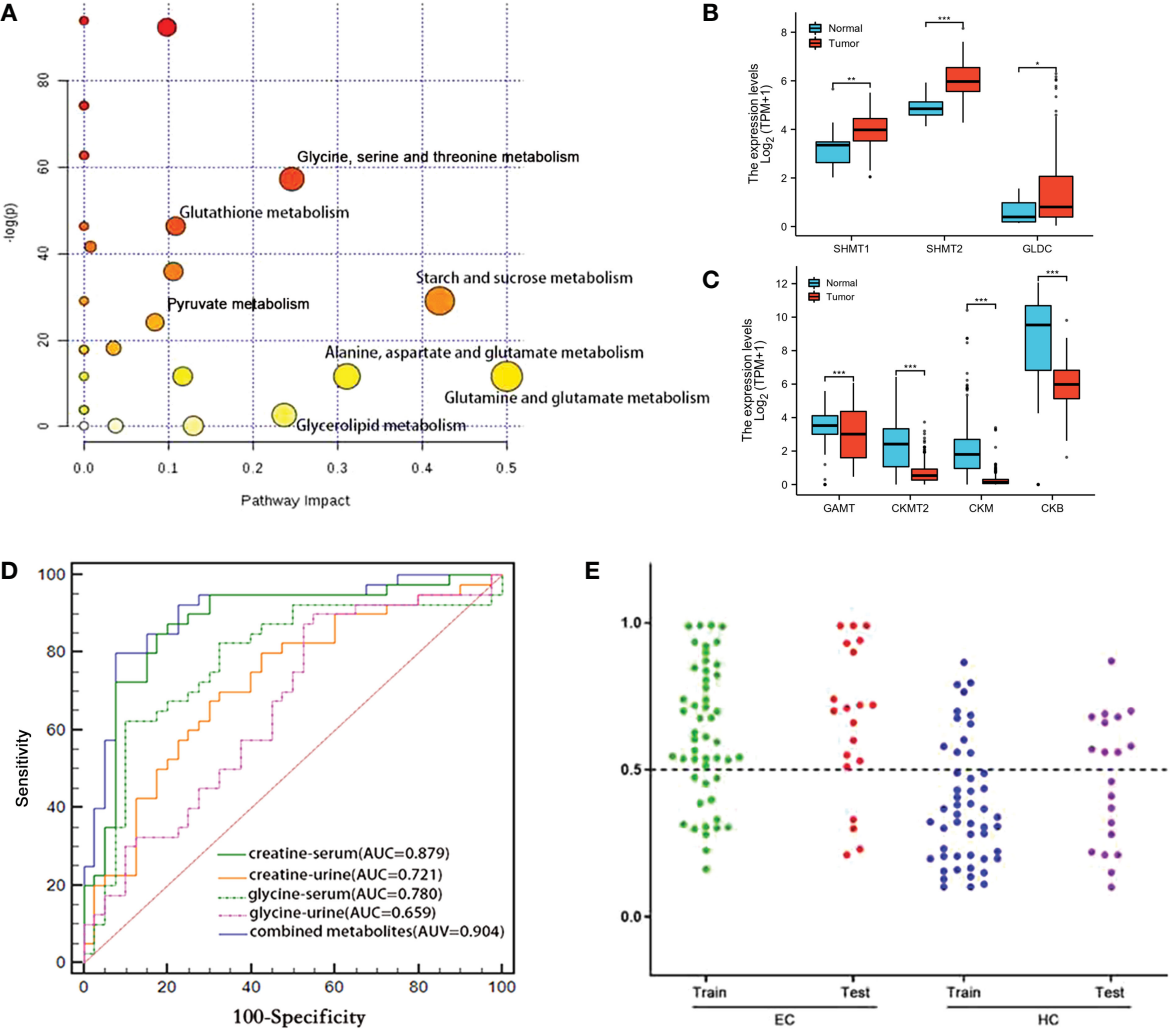
**(A)** Metabolic pathways associated with various metabolic alterations in ESCC vs. HCs. **(B, C)** Comparison of the metabolic enzyme gene expression related to “glycine, serine and threonine metabolism” between ESCC cancer tissues and normal tissues (ns, p≥0.05; *, p< 0.05; **, p<0.01; ***, p<0.001). **(D)** Comparison of the ROC curves used for distinguishing ESCC patients from HCs, based on the levels of various individual serum and urine metabolites and a combined set of metabolites. **(E)** Prediction and validation of the diagnostic accuracy of the optimal combined biomarker panel for ESCC detection (“training set”: ESCC=50, HC=50; “discovery set”: ESCC=20, HC=20). ns, not statistically significant.

The obtained differential metabolites between the ESCC cancer tissues and DNT were subjected to MetaboAnalyst 5.0 to determine the important disturbed pathways, with the pathway impact value≥ 0.1 and the –log(p) value≥2. As can be seen in **Figure 4** **A**), “Pyruvate metabolism”, “Glutathione metabolism”, “Glycine, serine and threonine metabolism” and “Starch and sucrose metabolism” were the significant pathway alterations associated with ESCC progression.

According to the analysis of TCGA-ESCA data, we found that the expression of serine hydroxymethyltransferases 1, 2 (SHMT1, SHMT2) and glycine decarboxylase (GLDC), which catalyze the conversion of serine to glycine, were upregulated in ESCC cancer tissues compared with normal tissues (**Figure 4B**). However, guanidinoacetate N-methyltransferase (GAMT), which converts guanidinoacetate to creatine, was downregulated (**Figure 4C**). Meanwhile, subsequent reduction of creatine leads to reduced levels of creatine kinases, including CKMT2 (Creatine kinase S-type, mitochondrial), CKM (Creatine kinase M-type) and CKB (Creatine kinase B-type). The alterations of metabolic enzyme gene expression mentioned above in TCGA-ESCA database were consistent with the elevated glycine and decreased creatine in ESCC cancer tissues we observed.

Given that glycine and creatine were the overlapping discriminative metabolites across serum, urine and cancer tissues in ESCC patients, and also they were the major metabolites involved in the perturbation of “glycine, serine, and threonine metabolism”, the significant pathway alteration associated with ESCC progression, an optimal biomarker panel was therefore selected by combining glycine and creatine in both serum and urine. Compared to the individual biomarker of glycine and creatine in serum or urine, this panel demonstrated superior diagnostic performance to detect ESCC, with a sensitivity, specificity and an AUC value of 80.0%, 92.5% and 0.904, respectively (**Figure 4D**). To further verify this panel’s reliability in detecting ESCC, we established a logistic model based on the test group (ESCC=50, HC=50) to predict the profiles of urine and serum samples in the validation group (ESCC=20, HC=20). The diagnostic accuracy of the test group and validation group were 72% and 80%, respectively (**Figure 4E**), which were significantly higher than that of CEA (58%) and CA19–9 (48%) (**Table 1**).

### Nomogram construction to predict the risk of ESCC

**Figure 5:**
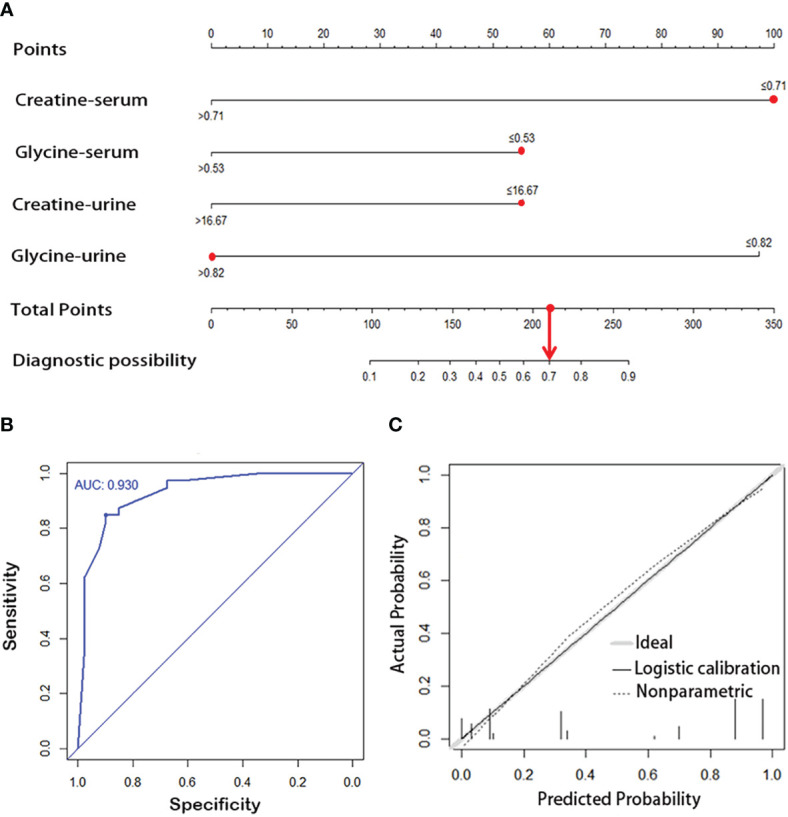
**(A)** Visual predictive nomogram model based on serum and urine metabolic signature of ESCC patients. The No. 1 sample in the modeling group has a total score of 210 points, and the risk of ESCC is 70%. ROC curve **(B)** and calibration cure **(C)** of the nomogram model for predicting esophageal cancer.

Glycine and creatine in both serum and urine were subjected to Logistic single-factor and multi-factor regression analysis, and a logistic regression model was constructed, which was visualized by R language software as a predictive nomogram diagnostic scoring model (**Figure 5** **A**), with the score of each variable ranging from 0 to 100. Total scores can be obtained by calculating the scores of glycine and creatine in both serum and urine, which were then projected onto the lower overall scale axis, with lower scores indicating a lower risk of ESCC. As can be seen in **Figure 5** **A**, the modelling group No. 1 sample had a total score of 210 points, and the risk of ESCC was 70%. Endoscopy was suggested and confirmed that the No.1 sample was an early esophageal cancer. The diagnostic efficiency of this predictive nomogram was higher than any diagnostic model constructed by a single serum or urine metabolic biomarkers, evidenced by a good prediction ability of 93% for ESCC detection (**Figure 5** **B**), and the prediction curve in the calibration graph and the standard curve fitted well (**Figure 5** **C**).

## Discussion

The diagnosis of esophageal cancer (more than 90% of which are ESCC) is mainly based on the clinical symptoms and invasive pathological examination, which lags far behind the disease progression and has limited role in cancer early diagnosis. Non-invasive, simple and accurate biomarkers may be more suitable for population screening. ^1^H-NMR-based metabolomics provides simple, efficient and inexpensive technical support for detecting the metabolic fingerprints of ESCC. In this study, we identified significant NMR-based metabolic alterations in serum, urine and tumor tissues in ESCC patients compared to their respective controls, and significant alterations of many metabolites in serum and urine were linked to the metabolic profiles of ESCC cancers tissues. Creatine and glycine in both serum and urine were selected as the potential biofluid biomarker panel for ESCC detection as they were the overlapping discriminative metabolites across serum, urine and tissue samples in ESCC patients, and also they were the major metabolites involved in the perturbation of “glycine, serine, and threonine metabolism”, the significant pathway alteration associated with ESCC progression. Then, a predictive nomogram model was constructed by combining creatine and glycine in both serum and urine (**Figure 5A**), which exhibited an improved diagnostic efficiency of ESCC (with an AUC of 0.930), and the predicted curve in the calibration chart fit well with the standard curve (**Figures 5B, C**). Overall, changes of serum and urine metabolism in ESCC patients can reflect the characteristics of metabolic disorders in tumor tissues, emphasizing the potential utility of NMR-based biofluids metabolomics fingerprinting as a non-invasive predictor for ESCC detection. Our study has several strengths. First, we systematically analyzed the ESCC cancer tissues and distant non-cancerous mucosa, as well as preoperative serum and urine samples from the same ESCC patients, to observe the common malignancy-related metabolic profiles in three samples. Second, *in situ* metabolic analysis based on ESCC cancer tissues was used as an internal standard to determine tumor-related metabolic biomarkers overlapping in serum and urine. Third, a visual predictive nomogram model based on tumor-specific metabolic profiles overlapping in serum and urine was constructed to improve the diagnostic efficiency of ESCC.

### ESCC-related metabolomic alterations in serum, urine and cancer tissues

**Figure 6:**
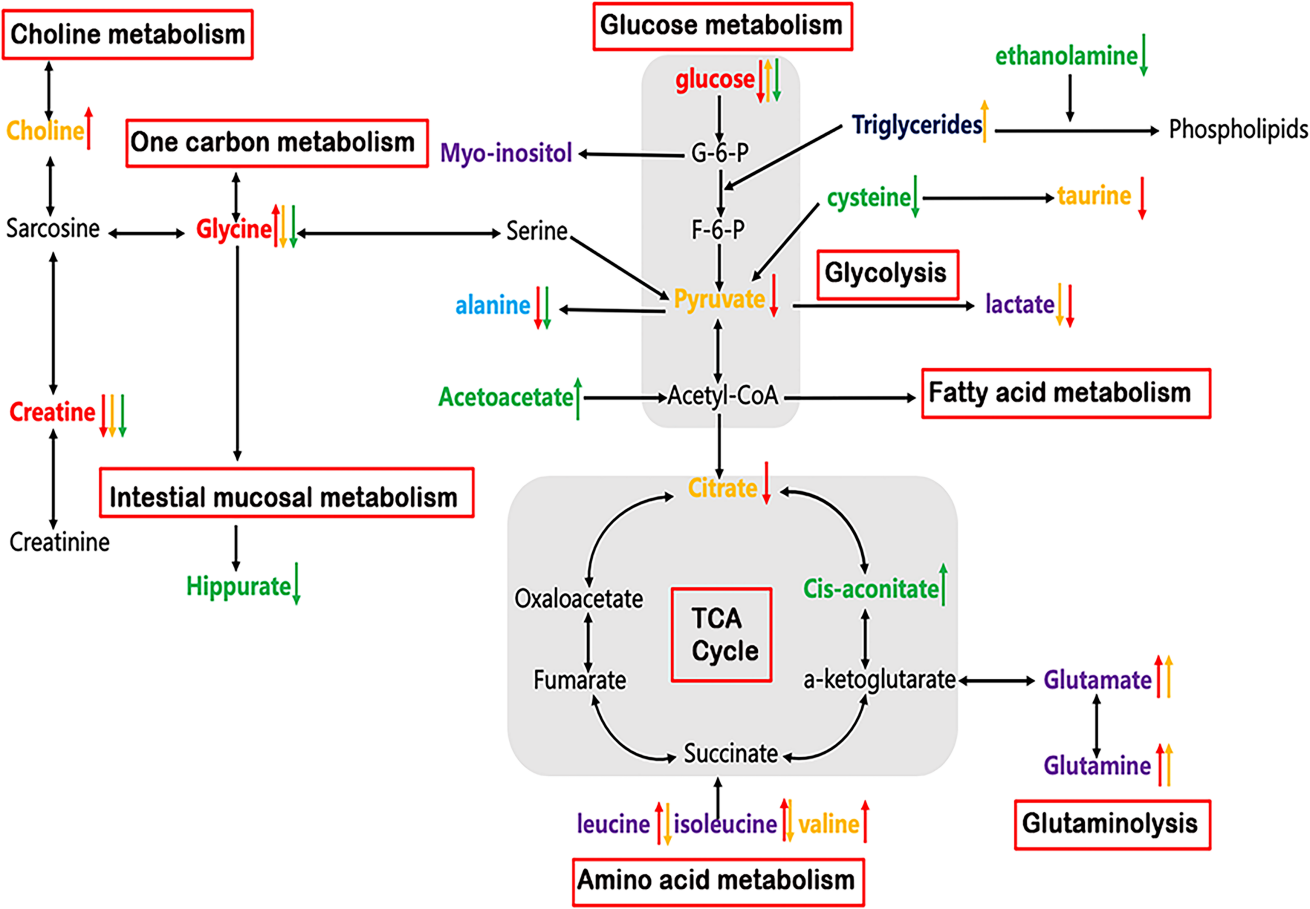
Altered metabolic pathways for the most relevant distinguishing metabolites (Red: common changed metabolites in tissue, serum and urine; Purple: changed metabolites in both tissue and serum; Light blue: changed metabolites in both tissue and urine; Yellow: tissue-specific metabolites; Navy: serum-specific metabolites; Green: urine-specific metabolites. Red arrow: increased or decreased with respect to control in tissue; Yellow arrow: increased or decreased with respect to control in serum; Green arrow: increased or decreased with respect to control in urine).

In addition to genetic and proteomic alterations, ESCC development is in association with cellular metabolic alterations that can give discernment into disease pathogenesis (22–24). In this study, significant alterations of many metabolites in serum, urine and tumor tissues were identified in ESCC patients compared to their respective controls (**Table 2**), indicating a potential network of metabolic pathway disturbances, such as TCA cycle, glycolysis, gluconeogenesis, glutaminolysis, amino acid metabolism, one-carbon unit metabolism, etc (**Figure 6**).

As can be seen in **Table 2**, reduced glucose together with depleted citrate (TCA intermediates) were observed in ESCC tumor tissues, reflecting an enhanced activation of glycolysis, which increases the demand and utilization of glucose and TCA intermediates to promote tumor growth (25, 26). Lactate was previously thought to be only a metabolic waste product of glucose metabolism. However, in recent years, more and more studies have demonstrated that lactate can promote tumor progression. For example, high concentrations of lactate are delivered into cells to be metabolized as a fuel substrate (27). Here, we did not observe an elevation of lactate in ESCC cancer tissues compared to DNT, which may imply that it is transported into cells as a fuel substrate. Increased glucose accompanied by decreased lactate in serum in ESCC patients indicated that the metabolic pathway of gluconeogenesis was enhanced to make up for the glucose consumed by the tumor cells (25, 26). In addition, we observed an increase in glutamate and a decrease in glutamine in the tissues and serum of ESCC patients, suggesting enhanced glutaminase activity for tumor development (28). Acetoacetate can be converted from fatty acids to acetyl-CoA, and enters TCA cycle to generate a large amount of ATP. Here, the levels of urine acetoacetate in ESCC patients increased, along with the elevated cis-aconitate (TCA intermediate), indicating that the TCA cycle pathway was active to provide energy for tumor cell proliferation (29). Choline is an important component in maintaining the integrity of cell membrane structure and function, and its metabolism is usually increased in malignant tumors (30, 31). Thus, the increased choline content observed in ESCC cancer tissues suggests that membrane biosynthesis was activated in response to the rapid tumor cells proliferation. The significant decrease of myo-inositol level in ESCC cancer tissues may be related to the decreased osmoprotective effect of cancer cells. Finally, we observed taurine depletion in ESCC cancer tissues, indicating intracellular injury and inflammatory response associated with ESCC (32).

Cancer cells typically exhibit high rates of anabolic metabolism, by which they absorb large amounts of nutrients to promote TCA cycling and oxidative phosphorylation. Thus, in addition to energy metabolism, tumor cells exhibit changes in nutrients to meet the increased amino acid metabolism for proliferation. Therefore, the amounts of amino acids, such as isoleucine, valine, leucine, arginine, lysine, and asparagine in ESCC cancer tissues, were relatively higher than those in the distant non-cancerous tissues, implying an active protein synthesis and amino acid metabolism in tumor tissues. This is because they are branched-chain amino acids that can be synthesized by condensation of the corresponding keto acids to promote protein biosynthesis. In addition, they can enter the tricarboxylic acid cycle through succinyl-CoA to promote energy metabolism (33, 34). The observed alanine depletion in ESCC cancer tissues is incordance with the use of ingested nutrients to promote TCA cycling.

Glycine and creatine were overlapping discriminatory metabolites across serum, urine and tissue samples in ESCC patients in this study. Besides, they were the main metabolites involved in the perturbation of glycine, serine and threonine metabolism, the most significant pathway alteration, suggesting that the serine/glycine synthesis and one-carbon unit metabolism pathways were disturbed, so as to maintain the malignant hyperplasia of the tumor. One-carbon unit is a key metabolic pathway for cell proliferation, mainly derived from the metabolism of glycine, serine, histidine and tryptophan. Its main function is to participate in the synthesis of purine-pyrimidine, choline and epinephrine, as well as the synthesis and modification of DNA and RNA (35, 36). The glycine level was found to be increased in tumor tissues and decreased in serum and urine in ESCC patients. The possible reason is that glycine in both serum and urine is delivered to the tissues, reducing their levels to supplement the glycine supply in the tissue, such producing a large amount of one-carbon to maintain the rapid proliferation of tumor tissue cells. The levels of creatine in ESCC cancer tissues and biofluids were lower than those in the normal controls, and the levels of creatine in serum and glycine in tissues were negatively correlated. This indicated that the metabolic pathway of glycine biosynthesis to provide one-carbon is enhanced and the metabolic conversion to sarcosine is weakened, thereby reducing the production of creatine. On the other hand, reduced creatine is associated with the changes in the process of energy transfer (37), suggesting that the activity of creatine phosphokinase is enhanced, and creatine is consumed to produce more phosphocreatine to meet the needs of hypermetabolism (38, 39). In addition, complementary data of enzyme genes related to serine and threonine metabolic pathway were analyzed (**Figures 5** **B, C**). Up-regulation of metabolic enzymes SHMT1, SHMT2 and GLDC, which catalyzes the conversion of serine to glycine (40) may result in increased glycine biosynthesis, and down-regulation of GAMT genes, using S-adenosylmethionine as the methyl donor to convert guanidinoacetate to creatine (41) may be associated with decreased creatine levels in ESCC cancer tissues.

### Construction of a predictive nomogram based on creatine and glycine in both serum and urine for ESCC detection

Creatine and glycine in both serum and urine were selected as the optimal biomarker panel for ESCC detection based on the following criteria: (i) they were the overlapping discriminative metabolites across serum, urine and tissue samples in ESCC patients; (ii) they were the major metabolites involved in the disturbance of “glycine, serine, and threonine metabolism”, the important metabolic pathway perturbation associated with ESCC progression; and (iii) changes in their biofluid levels were associated with changes of key genes expression involve in “glycine, serine, and threonine metabolism” in ESCC cancer tissues. Then, a predictive nomogram model was constructed by combining creatine and glycine in both serum and urine, which improved the diagnostic efficiency of ESCC compared to individual serum or urine metabolic biomarkers.

Nomogram is a mapping prediction model that comprehensively analyzes multiple quantitative and qualitative variables to predict the occurrence of a specific event. It can use intuitive mapping to assess the risk of individual patients. The model can be based on Logistic regression model and Cox regression model, and its results can be visualized. Specifically, the scoring standard is formulated according to the size of the model regression coefficient, and a score is then assigned to each value of each independent variable to calculate the total score of each patient. Subsequently, the probability of outcome occurrence of each patient can be calculated through the conversion function between the score and the probability of outcome occurrence. Its axis structure and risk points reflect the impact and importance of each variable on the prediction results. Nomogram is a practical and convenient tool for clinical application, which has been widely used in the research in tumor prediction, lymph node metastasis, prognosis, and efficacy judgment (42–44). The most crucial benefit of nomogram is that it can assess the risk in advance before the tumor progresses to the middle and advanced stage, and help clinicians to decide appropriate management methods, such as endoscopic follow-up intervals or selection of proper surgical procedures. As can be seen in **Figure 5** **A**, the modelling group No. 1 sample had a total score of 210 points, and the corresponding prediction probability of ESCC was 70%, which suggests that further clinical examinations, such as esophageal endoscopy is required. Indeed, endoscopy confirmed that the No.1 sample was an early ESCC.

### Limitations of this study

This study has limitations, including a small sample size, a limited number of precancerous lesions and tumors at each stage, lack of genomics-derived molecular features or validation of the metabolic pathway disorders at other systemic biological levels, which need to be studied and analyzed in the near future.

## Conclusion

Our paralleled investigation observed a considerable number of changed metabolites in ESCC cancer tissue, serum and urine, demonstrating the networks for metabolic pathway disturbance in ESCC subjects. The changes in urine and serum metabolism in esophageal cancer could reflect the metabolic disorders of the cancer tissues, highlighting that NMR-based biofluids metabolomics fingerprints, as non-invasive predictors, have the potential utility for ESCC detection. The visual predictive nomogram model based on creatine and glycine in both serum and urine could improve the diagnostic efficiency of esophageal cancer. Further studies based on a larger number size and in combination with other omics or molecular biological approaches are needed to validate the metabolic pathway disturbances in ESCC patients.

## Data availability statement

The raw data supporting the conclusions of this article will be made available by the authors, without undue reservation.

## Ethics statement

The studies involving human participants were reviewed and approved by The Ethical Review Board of Shantou University Medical College. The patients/participants provided their written informed consent to participate in this study.

## Author contributions

TO: Investigation, Data curation, Writing- Original draft preparation. CM: Investigation, Methodology, Resources. YZ: Investigation, Methodology, Resources. WY: Investigation. JZ: investigation. RC: Investigation. HZ: Investigation, Resources. PZ: Investigation. YL: Conceptualization, Methodology, Writing- Reviewing and Editing. The work reported in the paper has been performed by the authors, unless clearly specified in the text. All authors contributed to the article and approved the submitted version.
